# Socioeconomic factors and COVID-19 mortality in immune-mediated rheumatic diseases patients: regional analysis from Argentina, Mexico and Brazil

**DOI:** 10.1186/s42358-024-00418-3

**Published:** 2024-10-09

**Authors:** Marco Ulises Martinez-Martinez, Carolina Ayelen Isnardi, Deshiré Alpizar-Rodriguez, Guillermo Javier Pons-Estel, Belén María Virasoro, María Agustina Alfaro, Ingrid Petkovic, Rosana Quintana, Guillermo Berbotto, María Jezabel Haye Salinas, Sofía Ornella, Mariana Pera, Iris Jazmín Colunga-Pedraza, Fedra Irazoque-Palazuelos, Greta Reyes-Cordero, Tatiana S Rodriguez-Reyna, Jose Antonio Veloz-Aranda, Cassandra Michele Skinner-Taylor, Ingrid Maribel Juárez-Mora, Luis H. Silveira, Claudia Diniz Lopes Marques, Ricardo Machado Xavier, Adriana Maria Kakehasi, Ana Paula Gomides, Edgard Torres dos Reis-Neto, Gecilmara Salviato Pileggi, Gilda Aparecida Ferreira, Licia Maria Henrique da Mota, Marcelo Medeiros Pinheiro, Débora Cerqueira Calderaro

**Affiliations:** 1 Hospital General de Subzona No. 9, Instituto Mexicano del Seguro Social, San Luis, Potosí México; 2 Research Unit, Argentine Society of Rheumatology, CABA, Argentina; 3 Research Unit, Colegio Mexicano de Reumatología, Mexico City, Mexico; 4 Investigator of the SAR-COVID Registry, Argentine Society of Rheumatology, CABA, Argentina; 5 Sanatorio Petkovic, Tunuyán, Mendoza Argentina; 6 6Centro Regional de Enfermedades Autoinmunes y Reumáticas (GO—CREAR), Rosario, Santa Fe Argentina; 7 Sanatorio Británico Rosario, Chief of the Rheumatology Service, Rosario, Santa Fe Argentina; 8 Reumatologa CEMMA, Universidad Nacional de La Rioja, La Rioja, Argentina; 9 HIGA San Martin, La Plata, Buenos Aires Argentina; 10 Hospital Angel C Padilla, San Miguel de Tucuman, Tucuman, Argentina; 11 Investigator of CMR-COVID Registry, Mexican College of Rheumatology, Mexico City, Mexico; 12 Hospital Universitario José Eleuterio González, Monterrey, Mexico; 13 Centro de Investigación y Tratamiento Reumatológico S.C., Rheumatology, Mexico City, Mexico; 14 Facultad de Medicina y Ciencias Biomédicas, Universidad Autónoma de Chihuahua, Chihuahua, Private Practice Rheumatology, Chihuahua, Mexico; 15 Department of Immunology and Rheumatology, Instituto Nacional de Ciencias Médicas y Nutrición Salvador Zubirán, Mexico City, Mexico; 16 Hospital Regional ISSSTE, Leon, Mexico; 17 Instituto Mexicano del Seguro Social, Internal Medicine, Mexico City, Mexico; 18 Instituto Nacional de Cardiología, Mexico City, Mexico; 19 ReumaCoV-Brasil, Brazilian Society of Rheumatology, São Paulo, Brazil; 20 Universidade Federal de Pernambuco, Recife, Pernambuco Brazil; 21 Universidade Federal do Rio Grande do Sul, Porto Alegre, Rio Grande do Sul Brazil; 22 Universidade Federal de Minas Gerais, Alfredo Balena Avenue, 190, Belo Horizonte, Minas Gerais CEP: 30130-100 Brazil; 23 UniCEUB, Brasília, Distrito Federal Brazil; 24 Universidade Federal de São Paulo, São Paulo, São Paulo Brazil; 25 Medical Sciences, Medical School, Universidade de Brasília, Brasília, Distrito Federal Brazil

**Keywords:** COVID-19, SARS-CoV-2 infection, Immune-mediated rheumatic diseases, Epidemiology, Regional socioeconomic factors

## Abstract

**Background:**

SARS-CoV-2 infection has become a major international issue, not only from a medical point of view, but also social, economic and political. Most of the available information comes from the United States, Europe, and China, where the population and the socioeconomic status are very different from Latin American countries. This study evaluates the effect of regional socioeconomic characteristics on mortality due SARS-CoV-2 infection in patients with immune-mediated rheumatic diseases (IMRD) from Argentina, Mexico and Brazil.

**Methods:**

Data from three national registries, SAR-COVID (Argentina), CMR-COVID (Mexico) and ReumaCoV-Brasil (Brazil), were combined. Adult IMRD patients with SARS-CoV-2 infection were recruited. National data for each province/state, including population density, number of physicians per inhabitant, income, unemployment, GINI index, Municipal Human Development Index (MHDI), stringency index, vaccination rate and most frequent viral strains per period were assessed as risk factors for mortality due to COVID-19.

**Results:**

A total of 4744 patients were included, 2534 (53.4%) from SAR-COVID, 1166 (24.6%) from CMRCOVID and 1044 (22.0%) from ReumaCoV-Brasil. Mortality due to COVID-19 was 5.4%. In the multivariable analysis, higher number of physicians per 1000 inhabitants and being infected during the vaccination period of each country were associated with lower mortality. After adjustment for socioeconomic factors, there was no association with country of residence and mortality.

**Conclusion:**

These findings corroborate the complex interplay between socioeconomic factors, rheumatic disease activity, and regional disparities as determinants of death due to COVID-19 in Argentina, Brazil and Mexico. Thus, this research provides valuable insights for guiding public health policies and clinical practice in the ongoing fight against the COVID-19 pandemic.

**Supplementary Information:**

The online version contains supplementary material available at 10.1186/s42358-024-00418-3.

## Background

Host, viral and environmental factors have been associated with severe clinical outcomes of COVID-19. A large body of evidence supports the idea that age, ethnicity, sex, comorbidities, immunological history, viral factors and low socioeconomic status (SES) are risk factors for severe COVID-19 outcomes [1].

A recent meta-analysisof patients with rheumatic diseases reported a COVID-19 mortality rate of 0.0346 (95% CI: 0.0218–0.0493). This study also highlighted significant regional differences in mortality rates, with the following rates observed 0.0000 (95% CI: 0.0000–0.0202) in Asia, 0.0539 (95% CI 0.0295–0.0828)in Europe, 0.0477 (95% CI: 0.0345–0.0627) in North America, and 0.0838 (95% CI: 0.0536–0.1161) in South America (*p* < 0.05) [2].

The impact of COVID-19 has been particularly pronounced in Latin America and the Caribbean, accounting for 25% of SARS-CoV-2 infections globally [3]. In addition, great variability in the effect of the pandemic has been observed throughout this region. This can be attributed, at least in part, to differences in healthcare infrastructure, political leadership, poverty, and inequality, as well as variability in the country’s strategies to face the pandemic and access to vaccines [4].

In a study from the COVID-19 Global Rheumatology Alliance (C19-GRA) physician-reported registry, in which 14 044 patients with immune-mediated rheumatic diseases (IMRD) patients from 23 countries were included, the number of hospital beds (0·94 per 1-unit increase per 1000 people [0·88–1·00]; *p* = 0·046), human development index (0·65 per 0·1-unit increase [0·44–0·96]; *p* = 0·032), government response stringency (0·83 per 10-unit increase in containment index [0·74–0·93]; *p* = 0·0018), as well as follow-up time (0·78 per month [0·69–0·88]; *p* < 0·0001) were independently associated with lower odds of mortality. These factors may have contributed to the observed country-level variations in mortality rates (intraclass correlation coefficient 1·2% [0·1–9·5]; *p* = 0·14) [5].

The COVID-19 pandemic has exposed a myriad of factors influencing COVID-19 outcomes, including socioeconomic status, healthcare infrastructure, and regional disparities. In a previous study conducted by our group, patients with IMRD from Mexico exhibited a significantly increased risk of death from COVID-19 [OR: 2.98 (95%CI: 2.02–4.44), *p* < 0.001) compared to patients from Argentina and Brazil, even after adjusting for other risk factors associated with severe COVID-19 outcomes [6]. This study aimed to explore the impact of socioeconomic status, healthcare infrastructure, and regional disparities on mortality outcomes of patients with IMRD and SARS-CoV-2 infection across these three Latin American countries. Aditionally it sought to evaluate how these factors contributed to the excessive mortality observed in Mexico.

## Methods

### Registry designs

In this observational study, individual-level data (demographics, characteristics of rheumatic disease, and comorbidities at the time of COVID-19 diagnosis) from the SAR-COVID, CMR-COVID and ReumaCoV-Brasil registries, from Argentina, Mexico and Brazil, respectively, were collected. Adult patients with rheumatic IMRD with SARS-CoV-2 infection registered from 13 August 2020 to 20 February 2022 in SAR-COVID, from 17 April 2020 to 10 February 2022 in CMR-COVID, and from 20 May 2020 to 30 October 2021 in ReumaCoV-Brazil, were included. The number of rheumatologists participating in each registry was 142 in Argentina, 68 in Mexico, and 90 in Brazil.

Details of the design of the SAR-COVID Registry, from Argentina, the CMR-COVID Registry, from Mexico, and the ReumaCoV-Brasil Registry, from Brazil have been described previously [6–9]. Briefly, SAR-COVID is a national, multicentre, observational, voluntary registry including adult patients with a rheumatic disease and confirmed SARS-CoV-2 infection. A total of 143 independent rheumatologists from all over Argentina have registered to participate. All variables were collected by self-report, clinical and laboratory examination and/or medical records review, performed by the rheumatologist during patient hospitalization due to COVID-19, or at the patient control visit (virtual or face-to-face) performed after SARS-CoV-2 infection, depending on availability.

Regarding CMR-COVID Registry: a total of 68 Mexican voluntary rheumatologists collected information of their patients with rheumatic diseases and diagnosis of COVID-19: all the data were sent to the Mexican College of Rheumatology, verified and included in a database from the Global Rheumatology Alliance (GRA-COVID).

ReumaCoV-Brasil is a multicentre, observational, prospective registry designed to monitor adult patients with IMRD with a confirmed diagnosis of COVID-19, according to the Brazilian Ministry of Health criteria.

Data were collected either via telephone call (preferred due to social distancing measures) or through face-to-face visits.

### COVID-19 diagnosis

In the SAR-COVID Registry only patients with a diagnosis of SARS-CoV-2 infection confirmed by a positive polymerase chain reaction (PCR) test for the SARS-CoV-2 virus from nasopharyngeal or oropharyngeal swab, or an antibody serology testing, were included.

Among patients from the CMR-COVID Registry, COVID-19 diagnosis was based on polymerase chain reaction (PCR), antibody serology testing, or metagenomic testing; computed tomographic scans; laboratory assays; or symptoms alone.

In the ReumaCoV-Brasil registry, COVID-19 diagnosis was based on symptoms alone (flu-like symptoms in people in close contact with laboratory confirmed COVID-19 patients), laboratory diagnosis (through PCR or rapid antigen tests for SARS-CoV-2 virus from nasopharyngeal or oropharyngeal swab, or antibody serology testing), or computed tomographic scans.

### Inclusion and exclusion criteria

In the three registries, eligible patients were those aged ≥ 18 years with a prior diagnosis of an IMRD, according to the American College of Rheumatology (ACR) or European Alliance of Associations for Rheumatology (EULAR) criteria. Patients with no immune-mediated disease, such as osteoarthritis and fibromyalgia, with missing data regarding place of residence or date of COVID-19, as well as those who did not wish to participate or presented missing data on the primary outcome and its possible determinants were not included in this analysis (Supplementary Fig. 1).

### Outcome

Primary outcome was mortality in IMRD patients with COVID-19 from Argentina, Mexico and Brazil.

### Study variables

The variables significantly associated with increased mortality in our previous study [6] were evaluated in this study. These variables included age, male sex, living in Mexico, presenting diabetes or chronic kidney disease (CKD), the number of comorbidities, type of IMRD categorized as inflammatory joint diseases (IJD), connective tissue diseases (CTD)/vasculitis and others, rheumatic disease activity at the time of the infection, and the use of glucocorticoids or Rituximab.

### Country and regional level socioeconomic, environmental and vaccination data

The population density, average monthly income rate, unemployment rate, Gini index, municipal human development index (MHDI), and number of physicians per 1000 inhabitants were assigned based on the region of origin for each patient. COVID-19 variant, vaccination availability, and stringency index were assigned according to the date of COVID-19 symptoms onset and the patient’s country of origin. Country and regional-level covariates considered to be associated with a higher individual’s probability of death from COVID-19 were obtained from publicly available sources (Table 1). They included socioeconomic data, healthcare infrastructure, poverty, inequality, prevalent COVID-19 strains across the ongoing course of the pandemic, the availability of vaccines and the stringency index from each country and their different regions. Continuous socioeconomic variables were converted into categorical variables based on data ranges, which were divided into two equal intervals.

**Table 1:** Data sources of country-level covariates

Country-level covariates	Argentina	Brazil	Mexico
**Population density** Population divided by land area, km^2^	INDEC, Censo 2022 https://www.indec.gob.ar/indec/web/Nivel4-Tema-2-41-165	IBGE, Censo 2022 https://www.ibge.gov.br/estatisticas/sociais/trabalho/22,827-censo-demografico-2022.html	INEGI, 2020 https://en.www.inegi.org.mx/app/tabulados/interactivos/?pxq=Poblacion_Poblacion_07_fb7d5132-39f0-4a6c-b6f6-4cbe440e048d
**Average monthly income** The average total amount of the money received in a month	INDEC https://www.indec.gob.ar/uploads/informesdeprensa/eph_total_urbano_ingresos_02_234C62770929.pdf	IBGE, Censo 2022 https://sidra.ibge.gov.br/tabela/7395#resultado	IMEC, Índice de Competitividad Estatal 2022 https://docs.google.com/spreadsheets/d/1-fsrrjPy9LLn1bbZPQeQEWEjROBJRs4S/edit?usp=share_link%26ouid=112277324755993974616%26rtpof=true%26sd=true
**Unemployment rate** The rate of the unemployed people in the labor force	INDEC https://www.indec.gob.ar/uploads/informesdeprensa/eph_total_urbano_ingresos_02_234C62770929.pdf	IBGE, Censo 2022 https://sidra.ibge.gov.br/Tabela/4093#resultado	INEGI https://www.inegi.org.mx/app/tabulados/default.html?nc=624
**Gini index** Measures the *inequality* of *income*. A Gini coefficient of 0 reflects perfect equality, while a Gini coefficient of 1 reflects maximal inequality among values	INDEC https://www.indec.gob.ar/uploads/informesdeprensa/eph_total_urbano_02_23FECDE7B871.pdf	IBGE, Censo 2022 https://sidra.ibge.gov.br/tabela/7435#resultado	https://www.coneval.org.mx/coordinacion/entidades/Paginas/Informes_Pobreza_Evaluacion_2020.aspx
**Municipal human development index** Evaluates human development through schooling, longevity and income	https://www.undp.org/sites/g/files/zskgke326/files/2022-11/PNUD_ElMapaDelDesarrollo_FINAL_1.pdf	http://www.atlasbrasil.org.br/ranking	https://www.undp.org/sites/g/files/zskgke326/files/2023-02/INFORME_PNUD_2022_electronico-Portadas.pdf
**Number of physicians/1000 inhabitants**	Observatorio Nacional de Recursos Humanos en Salud https://www.argentina.gob.ar/sites/default/files/informe_fdt_datos2019_vf-1.pdf	IBGE, 2019 https://agenciadenoticias.ibge.gov.br/agencia-noticias/2012-agencia-de-noticias/noticias/27,614-ibge-divulga-distribuicao-de-utis-respiradores-medicos-e-enfermeiros	IMEC, Índice de Competitividad Estatal 2022 https://docs.google.com/spreadsheets/d/1-fsrrjPy9LLn1bbZPQeQEWEjROBJRs4S/edit?usp=share_link%26ouid=112277324755993974616%26rtpof=true%26sd=true
**COVID-19 variant** Prevalent variant across the ongoing course of the pandemic	Our World in Data https://ourworldindata.org/covid-vaccinations	Our World in Data https://ourworldindata.org/covid-vaccinations	Our World in Data https://ourworldindata.org/covid-vaccinations
**Vaccine availability** COVID-19 vaccination availability across the ongoing course of the pandemic	Our World in Data https://ourworldindata.org/covid-vaccinations	Our World in Data https://ourworldindata.org/covid-vaccinations	Our World in Data https://ourworldindata.org/covid-vaccinations
**Stringency Index** Composite measure based on nine response indicators including school closures, workplace closures, and travel bans, rescaled to a value from 0 to 100 (100 = strictest)	Our World in Data https://ourworldindata.org/covid-vaccinations	Our World in Data https://ourworldindata.org/covid-vaccinations	Our World in Data https://ourworldindata.org/covid-vaccinations

*INDEC* Instituto Nacional de Estadística y Censo, *IBGE* Instituto Brasileiro de Geografia e Estatística, *INEGI* Instituto Nacional de Estadística y Geografía, *IMEC* Instituto Mexicano para la Competitividad

The Gini index measures the extent to which income or consumption distribution among individuals or households deviates from perfect equality. A Gini index of 0 represents perfect equality, while an index of 100 implies perfect inequality [10].

The stringency index is a composite measure based on nine response indicators including school closures, workplace closures, and travel bans, rescaled to a value from 0 to 100 (100 = strictest) [11]. The most recent data available up to the period the registers were retrieved.

### Ethical considerations

The protocols from all three registries and their corresponding informed consent forms were approved by local independent ethics committees.

### Statistical analysis

Descriptive analysis was carried out. Socioeconomic variables, including range of physicians per 1000 inhabitants, average monthly income, Gini index, unemployment rate, MHDI and population density exhibited a non-normal distribution. They were dichotomized into two levels based on their respective ranges. Patients who experienced the onset of COVID-19 symptoms during the period of vaccination were categorized under the “vaccination period,” while those who did not were classified under the “non-vaccination period” for each respective country.

To establish the association between death related to COVID-19 and the different demographic and clinical factors, Chi-square test was used for categorical variables, and if assumptions were not fulfilled, categories were grouped applying Fisher’s exact test. For continuous variables with normal distribution, Student t test was used. In case of violation of the assumption of normality, the Mann-Whitney nonparametric test was applied.

Based on the socioeconomic data collected for each region, Euclidean distance was evaluated, and a cluster analysis was conducted. According to the statistical analysis, two clusters were suggested. Cluster 1 comprised all regions from Mexico and Brazil, while Cluster 2 encompassed all regions from Argentina. The data were then visualized using a heatmap based on Euclidean distances.

A logistic regression analysis was also performed to investigate the associations among mortality and regional sociodemographic and clinical variables. The initial “saturated” logistic regression model included the significant variables from our previous study [6] (country of origin, age, sex, current use of glucocorticoids or rituximab, having diabetes or CKD, IMRD activity) and regional socioeconomic variables (physicians/1000 inhabitants, average monthly income, Gini index, unemployment rate, MHDI, population density, if the patient was infected by COVID-19 in the vaccination period or not, predominant variant in the inclusion period and the stringency index). Adjustment for confounding variables was carried out. The absence of collinearity among all the variables included in the logistic regression model development was ensured. Trends of variables were explored using non-parametric regression models, which demonstrated linearity of age. Our initial model comprised all variables, and subsequently, we eliminated one by one any variables with a *p*-value less than 0.10. For multivariate analysis, missing data were imputed using multiple imputations with the “mice” package in R. To evaluate the effects of imputation, we conducted a sensitivity analysis of the logistic regression model.

Statistical analysis was conducted using R, version 4.3.2 (The R Foundation for Statistical Computing) and RStudio Version 2023.12.0 + 369 A *p*-value < 0.05 was considered statistically significant.

## Results

A total of 4744 patients with IMRD and SARS-CoV-2 infection were included, 2534 (53.4%) from SAR-COVID, 1166 (24.6%) from CMR-COVID and 1044 (22.0%) from ReumaCoV-Brasil. Overall, 82.3% were females with a mean age of 49.7 years old (SD 14.3). The most frequent IMRD was rheumatoid arthritis (43.2%), followed by systemic lupus erythematosus (22.1%). Half (55.3%) of the patients had IJD and over 70% of the IMRD were in remission or low disease activity at the time of SARS-CoV-2 infection diagnosis. The use of glucocorticoids was reported by 38.2% of the patients, 41.6% were receiving methotrexate and/or leflunomide and 14.8% were immunosuppressants. Moreover, 5.6% were treated with biologic DMARDs and 4.1% JAK inhibitors. A total of 43.7% of the patients had at least one comorbidity, with arterial hypertension and obesity being the most prevalent (Table 2).

**Table 2:** Characteristics of the IMRD patients with SARS-Cov-2 infection

	Argentina *n* = 2534	Brazil *n* = 1044	México *n* = 1166	*p*	Total *n* = 4744
Age, mean (SD)	50.1 (14.2)	47.0 (13.6)	51.2 (14.7)	<0.001	49.69 (14.28)
Male, *n* (%)	472 (18.6)	190 (18.2)	179 (15.4)	0.048	841 (17.7)
Use of glucocorticoids, *n* (%)	930 (36.7)	380 (36.4)	503 (43.1)	<0.001	1813 (38.2)
Use of rituximab, *n* (%)	63 (2.5)	37 (3.5)	64 (5.5)	<0.001	164 (3.5)
Diabetes, *n* (%)	167 (6.6)	127 (12.2)	154 (13.2)	<0.001	448 (9.4)
Chronic kidney disease, *n* (%)	49 (1.9)	61 (5.8)	39 (3.3)	<0.001	149 (3.1)
Rheumatic disease activity, *n* (%)				<0.001	
Remission	863 (36.0)	248 (33.1)	517 (48.6)		162 (3.8)
Low	1022 (42.7)	235 (31.3)	317 (29.8)		1574 (37.4)
Moderate	431 (18.0)	217 (28.9)	196 (18.4)		844 (20.1)
High	79 (3.3)	50 (6.7)	33 (3.1)		1628 (38.7)
Immune-mediated inflammatory disease, *n* (%)				<0.001	
IJD	1405 (55.4)	509 (48.8)	708 (60.7)		2622 (55.3)
CTD/Vasculitis	938 (37.0)	475 (45.5)	375 (32.2)		1788 (37.7)
Other diseases	191 (7.5)	60 (5.7)	83 (7.1)		334 (7.0)
Number of comorbidities, *n* (%)				<0.001	
0	1617 (63.8)	410 (39.3)	645 (55.3)		2672 (56.3)
1	492 (19.4)	367 (35.2)	345 (29.6)		1204 (25.4)
2	282 (11.1)	202 (19.3)	131 (11.2)		615 (13.0)
3	101 (4.0)	49 (4.7)	35 (3.0)		185 (3.9)
≥4	42 (1.7)	16 (1.5)	10 (0.9)		68 (1.4)
Physicians per 1000 habitants (1.06,8.8], *n* (%)	2172 (85.7)	1042 (100.0)	1166 (100.0)	<0.001	4380 (92.4)
(8.85,16.6], *n* (%)	362 (14.3)	0 ( 0.0)	0 ( 0.0)		362 (7.6)
Average Income (USD per month) (169,437], *n* (%)	2164 (85.4)	606 (58.2)	343 (29.4)	<0.001	3113 (65.6)
(438,707], *n* (%)	370 (14.6)	436 (41.8)	823 (70.6)		1629 (34.4)
Gini index (0.297,0.421], *n* (%)	950 (37.5)	26 (2.5)	160 (13.7)	<0.001	1136 (24.0)
(0.422,0.548], *n* (%)	1584 (62.5)	1016 (97.5)	1006 (86.3)		3606 (76.0)
Unemployment rate (%) (1.56,10.1], *n* (%)	1673 (66.0)	118 (11.3)	1166 (100.0)	<0.001	2957 (62.4)
(10.2,18.8], *n* (%)	861 (34.0)	924 (88.7)	0 ( 0.0)		1785 (37.6)
MHDI (0.69,0.786], *n* (%)	0 ( 0.0)	424 (40.7)	878 (75.3)	<0.001	1302 (27.5)
(0.787,0.884], *n* (%)	2534 (100.0)	618 (59.3)	288 (24.7)		3440 (72.5)
Population density (hab/km^2^) (0.2, 3131.0], *n* (%)	2534 (100.0)	1042 (100.0)	713 (61.1)	<0.001	4289 (90.4)
(3131.1–6262.8] *n* (%)	0 ( 0.0)	0 ( 0.0)	453 (38.9)		453 (9.6)
SARS-CoV-2 infection during vaccination period, *n* (%)	1419 (56.0)	62 (5.9)	328 (28.1)	<0.001	2935 (61.9)
Predominant SARS-CoV-2 viral variant, *n* (%)				<0.001	
Delta	47 (1.9)	6 (0.6)	127 (10.9)		180 (3.8)
Gamma	582 (23.0)	34 (3.3)	10 (0.9)		626 (13.2)
Omicron	246 (9.7)	1 (0.1)	62 (5.3)		3629 (76.5)
Other variants	1659 (65.5)	1003 (96.1)	967 (82.9)		309 (6.5)
Stringency Index, mean (SD)	75.3 (17.7)	72.8 (10.8)	61.2 (21.3)	<0.001	71.28 (18.38)
Dead due to COVID-19, *n* (%)	101 (4.0)	44 (4.2)	110 (9.4)	<0.001	255 (5.4)

*n number, *SD* standard deviation, *IJD* inflammatory joint disease, *CTD* connective tissue disease, *USD* United State Dollar, *MHDI* Municipal Human Development Index, *hab* habitants

Socioeconomic status was as follows [Mean (SD)]: number of physicians/100,000 inhabitants: 4.4 (3.7); monthly average income (US$): 361.78 (126.29); Gini index: 0.45 (0.06); unemployment rate: 8.73 (3.52); MHDI: 0.81 (0.05); population density (hab/km^2^): 665.0 (1790.45).

Population density and average monthly income (in USD) were significantly higher in Mexico. The Gini index showed significant disparities among the countries, being lower (higher equality) in Argentina, followed by Mexico and Brazil (the greater disparity). Unemployment rates were significantly higher in Brazil, while the value of the MHDI was higher in Argentina.

Regarding healthcare infrastructure, the number of physicians per 1000 inhabitants was higher in Argentina. COVID-19 occurring during the vaccination period was more frequently reported in Argentina, followed by Mexico and Brazil. There were 2935 (62%) patientswho presented with COVID-19 before vaccines were available. The prevalence of different COVID-19 variants also differed significantly among the countries, with the Gama variant being most prevalent in Argentina, the Delta variant in Mexico, and Brazil reporting lower prevalence of the Omicron variant. Additionally, stringency indexes were heterogeneous, with Argentina presenting the highest score, followed by Brazil and Mexico (Table 2).

### Mortality due to COVID-19

Globally, mortality due to COVID-19 was 5.4%. Mexico reported the highest mortality (9.4%), followed by Brazil (4.2%) and Argentina (4.0%) (*p* < 0.001). Mortality according to the diagnostic work-up for COVID-19 is shown in supplementary Table 1. In the univariable analysis, a 6% increase in the odds of mortality per additional year of age was identified. Male gender (OR: 1.43, 95% CI: 1.05–1.92, *p* = 0.021), the presence of diabetes (OR: 3.33, 95% CI: 2.44–4.50, *p* < 0.001) or CKD (OR: 5.13, 95% CI: 3.33–7.69, *p* < 0.001), the increasing number of comorbidities, the diagnosis of CTD/vasculitis (OR: 1.34, 95% CI: 1.03–1.75, *p* = 0.032) and other rheumatic diseases (OR: 1.83, 95% CI: 1.17–2.79, *p* = 0.006), when compared to IJD, high rheumatic disease activity at the time of the infection (OR: 4.71, 95% CI: 2.92–7.44, *p* < 0.001), and the use of glucocorticoids (OR: 2.56, 95% CI: 1.98–3.32, *p* < 0.001) or rituximab (OR: 5.16, 95% CI: 3.33–7.69, *p* < 0.001) were associated with increased mortality.

Regarding regional socioeconomic factors, living in Mexico (OR: 2.51, 95% CI: 1.90–3.32, *p* < 0.001), increased population density (OR: 2.47, 95% CI: 1.77–3.40, *p* < 0.001) and higher average monthly incomes (OR: 1.75, 95% CI: 1.11–2.45, *p* < 0.001) were associated with increased odds of mortality. On the contrary, a higher number of physicians per habitants (OR: 0.43, 95% CI: 0.20–0.79, *p* = 0.014) and MHDI (OR: 0.63, 95% CI: 0.49–0.82, *p* = 0.001), being infected during the vaccination period (OR: 0.60, 95% CI: 0.45–0.79, *p* < 0.001) and the predominant circulation time frame of the gamma and omicron variants (OR: 0.44, 95% CI: 0.23–0.87, *p* = 0.016 and OR: 0.41, 95% CI: 0.18–0.90, *p* = 0.027, respectively) in each country were associated with lower mortality. The stringency index and the Gini index were not associated significantly with mortality (Table 3).

**Table 3:** Factors associated with mortality due to COVID-19. Uni- and multivariable analysis

Variables	Not dead *n* = 4489	Dead *n* = 255	Univariable analysis OR (95% CI, *p*)	Multivariable analysis OR (95% CI, *p*)
Country, *n* (%)				
Argentina (ref)	2433 (96.0)	101 (4.0)	–	–
Brazil	1000 (95.8)	44 (4.2)	1.06 (0.73–1.51, *p* = 0.752)	0.67 (0.41–1.07, *p* = 0.095)
México	1056 (90.6)	110 (9.4)	2.51 (1.90–3.32, *p*<0.001)	1.29 (0.82–2.01, *p* = 0.259)
Age, mean (SD)	49.1 (14.0)	60.3 (14.7)	1.06 (1.05–1.07, *p*<0.001)	1.07 (1.06–1.08, *p*<0.001)
Male sex, *n* (%)	782 (93.0)	59 (7.0)	1.43 (1.05–1.92, *p* = 0.021)	1.60 (1.13–2.23, *p* = 0.007)
Glucocorticoid use, *n* (%)	1660 (91.6)	153 (8.4)	2.56 (1.98–3.32, *p*<0.001)	2.02 (1.50–2.72, *p*<0.001)
Rituximab use, *n* (%)	130 (79.3)	34 (20.7)	5.16 (3.41–7.62, *p*<0.001)	4.82 (3.03–7.52, *p*<0.001)
Diabetes, *n* (%)	387 (86.4)	61 (13.6)	3.33 (2.44–4.50, *p*<0.001)	2.38 (1.67–3.34, *p*<0.001)
Chronic kidney disease, *n* (%)	118 (79.2)	31 (20.8)	5.13 (3.33–7.69, *p*<0.001)	4.22 (2.56–6.81, *p*<0.001)
Rheumatic disease activity, *n* (%)				
Remission (ref)	1556 (95.6)	72 (4.4)	–	–
Low	1518 (96.4)	56 (3.6)	0.80 (0.56–1.14, *p* = 0.213)	0.83 (0.59–1.17, *p* = 0.300)
Moderate	803 (95.1)	41 (4.9)	1.10 (0.74–1.63, *p* = 0.623)	1.10 (0.74–1.62, *p* = 0.641)
High	133 (82.1)	29 (17.9)	4.71 (2.92–7.44, *p*<0.001)	4.91 (2.92–8.14, *p*<0.001)
Immune-mediated inflammatory disease, *n* (%)				
IJD (ref)	2502 (95.4)	120 (4.6)	–	–
CTD/Vasculitis	1680 (94.0)	108 (6.0)	1.34 (1.03–1.75, *p* = 0.032)	1.98 (1.45–2.70, *p*<0.001)
Other Diseases	307 (91.9)	27 (8.1)	1.83 (1.17–2.79, *p* = 0.006)	2.58 (1.57–4.14, *p*<0.001)
Number of comorbidities, *n* (%)				
0 (ref)	2595 (97.1)	77 (2.9)	–	–
1	1133 (94.1)	71 (5.9)	2.11 (1.52–2.94, *p*<0.001)	
2	549 (89.3)	66 (10.7)	4.05 (2.87–5.70, *p*<0.001)	
3	162 (87.6)	23 (12.4)	4.78 (2.87–7.71, *p*<0.001)	
≥4	50 (73.5)	18 (26.5)	12.13 (6.62–21.44, *p*<0.001)	
Physicians per 1000 inhabitants, *n* (%)				
1.06–8.8 (ref)	4134 (94.4)	246 (5.6)	–	–
8.85–16.6	353 (97.5)	9 (2.5)	0.43 (0.20–0.79, *p* = 0.014)	0.23 (0.09–0.51, *p* = 0.001)
Average Income (USD per month), *n* (%)				
169 437 (ref)	2978 (95.7)	135 (4.3)	–	–
438–707	1509 (92.6)	120 (7.4)	1.75 (1.36–2.26, *p*<0.001)	1.64 (1.11–2.45, *p* = 0.014)
GINI index, *n* (%)				
0.297–0.421 (ref)	1082 (95.2)	54 (4.8)	–	–
0.422–0.548	3405 (94.4)	201 (5.6)	1.18 (0.88–1.62, *p* = 0.285)	
Unemployment (%), *n* (%)				
1.56–10.1 (ref)	2790 (94.4)	167 (5.6)	–	–
10.2–18.8	1697 (95.1)	88 (4.9)	0.87 (0.66–1.13, *p* = 0.289)	
MHDI, *n* (%)				
0.690–0.786	1208 (92.8)	94 (7.2)	–	–
0.787–0.884	3279 (95.3)	161 (4.7)	0.63 (0.49–0.82, *p* = 0.001)	
Population density (habitants/km^2^), *n* (%)				
(0.2, 3131.0] (ref)	4084 (95.2)	205 (4.8)	–	–
(3131.1–6262.8]	403 (89.0)	50 (11.0)	2.47 (1.77–3.40, *p*<0.001)	
SARS-CoV-2 infection during vaccination period, *n* (%)	1739 (96.1)	70 (3.9)	0.60 (0.45–0.79, *p*<0.001)	0.68 (0.49–0.93, *p* = 0.019)
Predominant SARS-CoV-2 viral variant, *n* (%)				
Delta (ref)	165 (91.7)	15 (8.3)	–	–
Gamma	602 (96.2)	24 (3.8)	0.44 (0.23–0.87, *p* = 0.016)	
Omicron	298 (96.4)	11 (3.6)	0.41 (0.18–0.90, *p* = 0.027)	
Other	3424 (94.4)	205 (5.6)	0.66 (0.39–1.18, *p* = 0.135)	
Stringency Index, mean (SD)	71.3 (18.4)	71.5 (18.7)	1.00 (0.99–1.01, *p* = 0.817)	

*ref reference, *IJD* inflammatory joint disease, *CTD* connective tissue disease, *USD* United State Dollar, *MHDI* Municipal Human Development Index

**Fig. 1:**
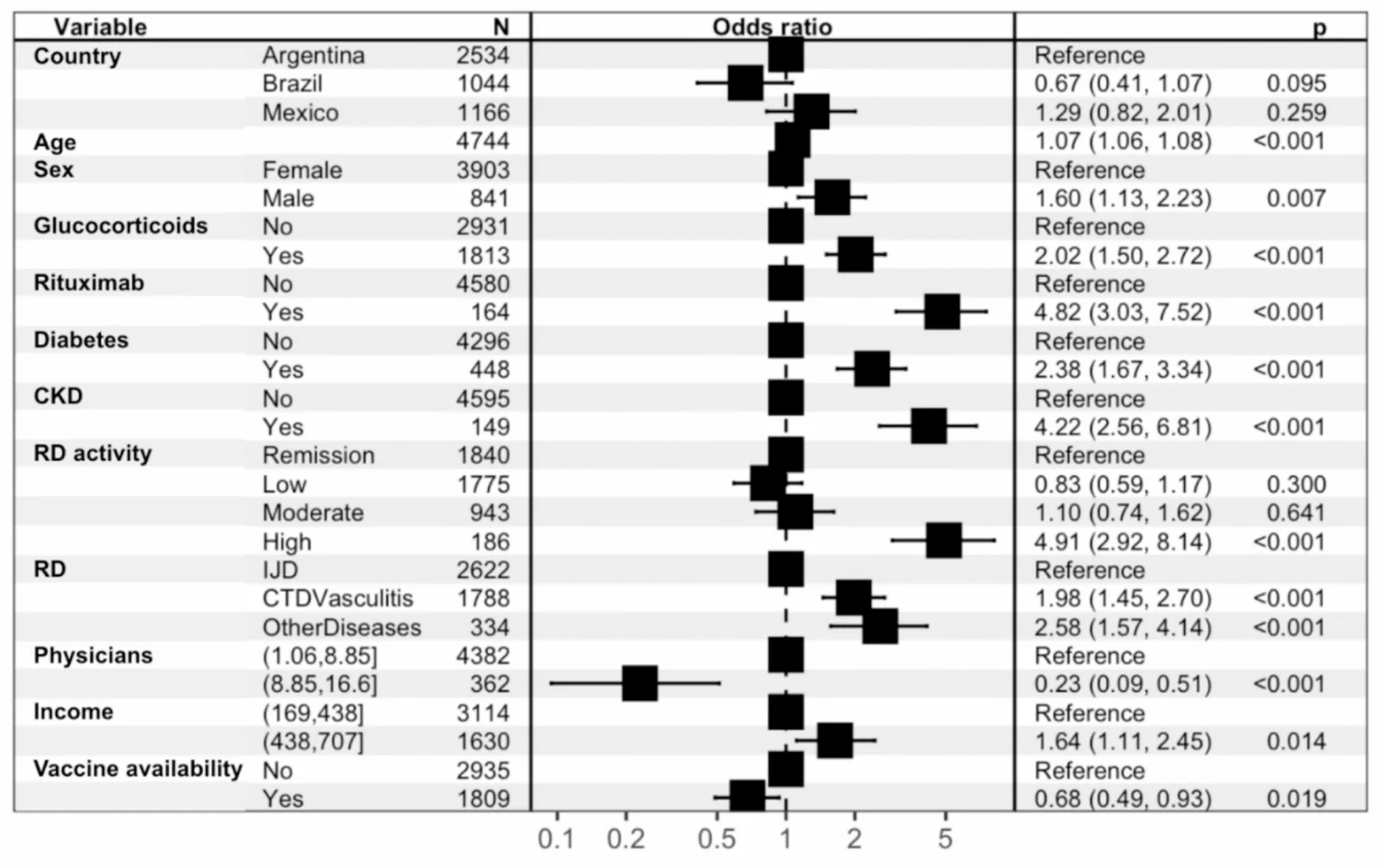
Factors associated with mortality. Multivariable analysis. Clinical and socioeconomic factors associated with mortality due to COVID-19 in patients with immune-mediated inflammatory diseases. Multivariable analysis after missing data imputation. *CKD Chronic kidney disease, *RD* Rheumatic disease, *IJD* Inflammatory joint disease, *CTD* Connective tissue disease

In the multivariable analysis, well-known risk factors maintained their statistical association with mortality, including older age, male sex, diabetes, CKD, type of IMRD, high rheumatic disease activity, the use of glucocorticoids and rituximab. After adjusting for confounders, higher number of physicians per 1000 inhabitants (OR: 0.23, 95% CI: 0.09–0.51, *p* = 0.001) and being infected during the vaccination period in each country (OR: 0.68, 95% CI: 0.49–0.93, *p* = 0.019) were associated with lower mortality, while higher average income remained associated with increased mortality (OR: 1.64, 95% CI: 1.11–2.45, *p* = 0,014) (Table 3; Fig. 1). No association between country of residence and mortality was established. After sensitivity analysis, including in the final model all variables with a *p*-value < 0.01, similar results were observed, except for the association between mortality and Gini index, which remained only with marginal significance (Supplementary Fig. 2).

### Cluster analysis

**Fig. 2:**
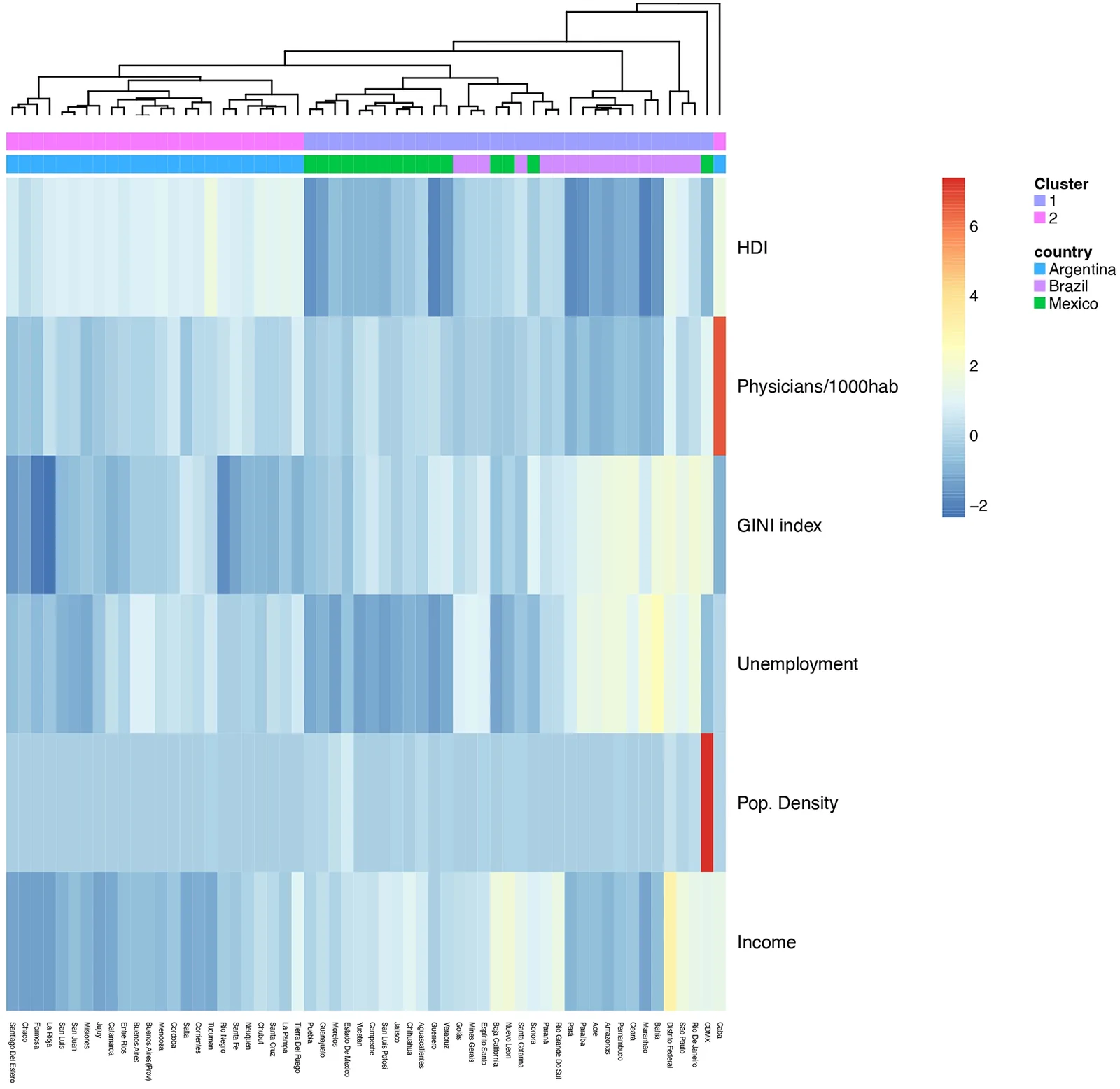
Clusters definition according to socioeconomic factors

Based on the socioeconomic data collected for each region of the three countries, two clusters were defined. According to this analysis, cluster 1 comprised all states from Brazil and Mexico, and cluster 2 included all provinces from Argentina (Fig. 2). Patients in cluster 1 were slightly younger, used glucocorticoids and rituximab more frequently and presented higher frequency of diabetes and CKD. In Argentina, provinces presented a higher density of physicians, MHDI, vaccine coverage and stringency index. On the contrary, lower population density, average income, Gini index, and unemployment rate were observed in this country. Gamma and omicron COVID-19 variants also were more frequent in Cluster 2. Cluster 1, including all regions from Mexico and Brazil, showed a significantly higher mortality than cluster 2 (7.0% vs. 4.0%, *p* < 0.001 (Table 4).

**Table 4:** Differences and similarities between clusters

	Cluster 1 (*n* = 2208)	Cluster 2 (*n* = 2534)	*p*-value
Country, *n* (%)			<0.001
Argentina	0 (0.0)	2534 (100.0)	
Brazil	1042 (47.2)	0 (0.0)	
México	1166 (52.8)	0 (0.0)	
Age, mean (SD)	49.2 (14.3)	50.1 (14.2)	0.033
Male, *n* (%)	368 (16.7)	472 (18.6)	0.078
Use of glucocorticoids, *n* (%)	882 (39.9)	930 (36.7)	0.022
Use of rituximab, *n* (%)	101 (4.6)	63 (2.5)	<0.001
Diabetes, *n* (%)	281 (12.7)	167 (6.6)	<0.001
Chronic kidney disease, *n* (%)	100 (4.5)	49 (1.9)	<0.001
Rheumatic disease activity, *n* (%)			<0.001
Remission	764 (42.2)	863 (36.0)	
Low	552 (30.5)	1022 (42.7)	
Moderate	413 (22.8)	431 (18.0)	
High	83 (4.6)	79 (3.3)	
Immune-mediated inflammatory disease, *n* (%)			0.256
IJD	1217 (55.1)	1405 (55.4)	
CTD/Vasculitis	849 (38.5)	938 (37.0)	
Other diseases	142 (6.4)	191 (7.5)	
Number of comorbidities, *n* (%)			<0.001
0	1054 (47.7)	1617 (63.8)	
1	711 (32.2)	492 (19.4)	
2	333 (15.1)	282 (11.1)	
3	84 (3.8)	101 (4.0)	
≥4	26 (1.2)	42 (1.7)	
Physicians per 1000 habitants (1.06,8.8], *n* (%)	2208 (100.0)	2172 (85.7)	
(8.85,16.6], *n* (%)	0 ( 0.0)	362 (14.3)	<0.001
Average Income (USD per month) (169,438], *n* (%)	949 (43.0)	2164 (85.4)	
(438,707], *n* (%)	1259 (57.0)	370 (14.6)	<0.001
Gini index (0.297,0.422], *n* (%)	186 ( 8.4)	950 (37.5)	
(0.422,0.548], *n* (%)	2022 (91.6)	1584 (62.5)	<0.001
Unemployment rate (%) (1.56,10.2], *n* (%)	1284 (58.2)	1673 (66.0)	
(10.2,18.8], *n* (%)	924 (41.8)	861 (34.0)	<0.001
MHDI (0.69,0.787], *n* (%)	1302 (59.0)	0 ( 0.0)	
(0.787,0.884], *n* (%)	906 (41.0)	2534 (100.0)	<0.001
Population density (hab/km^2^) (0.2, 3131.1], *n* (%)	1755 (79.5)	2534 (100.0)	<0.001
(3131.1–6262.8] *n* (%)	453 (20.5)	0 (0.0)	
SARS-CoV-2 infection during vaccination period, *n* (%)	390 (17.7)	1419 (56.0)	<0.001
Predominant SARS-CoV-2 viral variant, *n* (%)			<0.001
Delta	133 (6.0)	47 (1.9)	
Gamma	44 (2.0)	582 (23.0)	
Omicron	63 (2.9)	246 (9.7)	
Other variants	1968 (89.1)	1659 (65.5)	
Stringency Index, mean (SD)	66.7 (18.1)	75.3 (17.7)	<0.001
Dead due to COVID-19, *n* (%)	154 (7.0)	101 (4.0)	<0.001

*IJD inflammatory joint disease, *CTD* connective tissue disease, *USD* United State Dollar, *MHDI* Municipal Human Development Index, *hab* habitants

## Discussion

Our study evaluates the association of regional socioeconomic factors and healthcare resources with mortality in IMRD patients with COVID-19 from Latin America. It corroborates individual characteristics, such as older age, male sex, some comorbidities, especially diabetes mellitus and CKD, high rheumatic disease activity and immunosuppression with glucocorticoids or rituximab, and rheumatic diseases other than inflammatory joint diseases, are independent contributors to increased mortality. It also brings data on the positive impact of the regional physicians’ rates and vaccination decreasing mortality. Besides, our findings also suggest that regional socioeconomic factors may have contributed to the previous report of increased mortality from COVID-19 in IMRD patients from Mexico, when compared to Argentina and Brazil, since this excess subsided after adjustment for these data.

Evidence suggests that social disparities in COVID-19 mortality may reflect a combination of factors resulting in an increased vulnerability to being exposed to the virus, such as household crowding, underlying medical conditions, occupation and modes of transportation [12]. Additionally, inequities in healthcare infrastructure, exemplified by variations in the number of physicians per population and access to medical resources, likely contribute to differential mortality rates [13, 14]. For instance, regions with higher population density exhibited increased odds of mortality, possibly due to overwhelmed healthcare systems and limited access to critical care services [15]. In this context, in our study, cities with higher numbers of physicians showed a decrease in 80% odds of mortality.

A systematic review with meta-analysis that included 52 observational studies [16], mainly from the United States, suggested that racial/ethnic minority groups had higher risks of COVID-19 infection and hospitalization, confirmed diagnosis and death. Additionally, most of the studies described low levels of education, poverty, poor housing conditions, low household income, speaking in a language other than the national language and living in overcrowded households as risk factors for COVID-19 infection, death and confirmed diagnosis [5, 15, 16]. On the contrary, our data suggest that patients receiving over 440 USD per month showed poorer outcomes, with an increase of 64% of mortality probability. The authors believe the association between lower household income and lower mortality is probably due to an inclusion bias in the registers, since people from areas with lower household incomes probably had less access to COVID-19 testing, increasing the risk of underreporting of deaths related to this disease. Additionally, in this study, higher income rates were associated with locations exhibiting poorer Gini index distribution (indicating greater inequity), with a higher prevalence of diabetes, and a greater use of corticosteroids and rituximab. Therefore, it is not accurate to directly associate higher income with worse outcomes, as this income data was not obtained directly from the included patients. This contradictory result aligns with existing literature highlighting the complex interplay between socioeconomic status and health outcomes, where higher income may not necessarily confer better protection, particularly during pandemics [17–19].

Previous studies addressing access to SARS-CoV-2 tests presented controversial results. In one study from the United States (US) [20], the number of total tests was not associated with the socioeconomic status index, which was a composite score made of socioeconomic variables including household income, gross rent, poverty, education, working class, unemployment, and household density [21]. Another US study, however, showed that testing frequency was higher in areas with higher population density and public transportation volume, fewer uninsured individuals and more people with health access and higher per capita health care spending [22]. In Brazil, a positive association between higher per-capita income and COVID-19 diagnosis was previously suggested, while severe acute respiratory infection cases with unknown aetiology were associated with lower per-capita income, possibly due to the under testing and underreporting of COVID-19 in these patients [23].

COVID-19 represents a syndemic, synergistic condition that interacts with rheumatic diseases, comorbidities and socioeconomic factors [24–27]. One previous study from the C19-GRA physician-reported index, which evaluated the influence on mortality of some regional-level factors in 23 countries. A higher number of hospital beds, human development index and government response stringency index were associated with a decrease in COVID-19 mortality [5]. Additionally, a study from Argentina described socioeconomic factors related to social inequality, such as non-Caucasian (mestizo) ethnicity and public health insurance increased odds of hospitalization due to COVID-19 [28].

Moreover, a positive correlation between the levels of health inequity and mortality across neighbourhoods in the city of Buenos Aires and Bariloche have been reported [29, 30]. In São Paulo, less education, more household crowding and lower income were associated with higher mortality rates from COVID-19 [12]. A Brazilian nationwide study of healthcare registers showed that deprived municipalities presented increased odds of death as well as negative vaccination rates [31]. On the other hand, in Mexico, individuals living in municipalities with extreme poverty, social deprivation, limited access to health services and social security, high rates of crowding conditions, illiteracy and food insecurity presented an increased risk of dying from COVID-19 [32–35].

Moreover, cluster 1 exhibited a higher frequency of SLE but a lower frequency of vasculitis. This cluster also had significantly higher use of corticosteroids and rituximab, factors associated with increased mortality. However, these differences across the clusters could be attributed to variable factors that were not addressed in this study, including differences in healthcare access and prescription practices for rituximab among countries [36].

To the authors’ knowledge, this is the first work evaluating the association of regional socioeconomic factors and healthcare resources with mortality in IMRD patients with COVID-19 from Latin America. An important strength is the number of patients from three of the most populated Latin American countries with diverse cultural, socioeconomic and ethnic backgrounds. Our study adds to the current data a cluster analysis of regional sociodemographic, environmental factors, and healthcare resources, which helped understand the potential effect of these factors as mechanisms of disparate COVID-19 mortality in people with rheumatic diseases across clusters of nations sharing similarities.

Despite its strengths, this study has some limitations, including its observational design, which precludes causal inference, and potential confounding by unmeasured variables. Future research should explore additional determinants of COVID-19 outcomes, environmental exposures, and social determinants of health, to provide a more comprehensive understanding of disease disparities. Moreover, longitudinal studies are needed to assess the long-term impact of COVID-19 on patients with rheumatic diseases and inform tailored intervention strategies. There is also the possibility of a selection bias, favouring the inclusion of patients with more severe COVID-19, since most of them were symptomatic, had the COVID-19 diagnosis confirmed by a test, and attended rheumatology tertiary centres. There is also a tendency of reporting severe, hospitalized, and deceased cases. Other factors, such as limited access to diagnostic tests, may have contributed to the underreporting of asymptomatic or non-severe cases within this population. Unfortunately, data about glucocorticoid dose was not consigned in a homogeneous way between the registries and therefore this factor could not be analysed. Furthermore, during the onset of the pandemic, the lack of confirmatory testing may have contributed to the loss of some cases. Moreover, the registries did not include data on socio-demographic and economic characteristics, or information about access to health services and they did not address vaccination either. For this reason, socioeconomic data was collected regionally and according to the date of SARS-CoV-2 infection, but not individually.

In conclusion, this study underscores the multifactorial nature of COVID-19 outcomes in patients with rheumatic diseases and highlights the importance of addressing socioeconomic disparities and optimizing disease management strategies to mitigate mortality risks. By elucidating the complex interplay between socioeconomic factors, rheumatic disease activity, and regional disparities, this research provides valuable insights for guiding public health policies and clinical practice in the ongoing fight against the COVID-19 pandemic and the optimal care of people with rheumatic diseases.

## Electronic supplementary material

Below is the link to the electronic supplementary material.

Supplementary Material 1

Supplementary Material 2

Supplementary Material 3

## Data Availability

All data sets generated and analyzed, are available upon request to the corresponding author.
