# Supplementary material for: Socioeconomic factors and COVID-19 mortality in immune-mediated rheumatic diseases patients: regional analysis from Argentina, Mexico and Brazil
Source: Adv Rheumatol. 2024 Oct 9;64:78. doi: 10.1186/s42358-024-00418-3 (PMC13488799; doi:10.1186/s42358-024-00418-3)
Supplement: Supplementary file 2 [file 42358_2024_418_MOESM2_ESM.docx]

**Supplementary figure 2. Factors associated with mortality due to COVID-19. Multivariable analysis (No missing data imputation)**

Supplementary figure 2. Clinical and socioeconomic factors associated with mortality due to COVID-19 in patients with immune-mediated inflammatory diseases. Multivariable analysis without missing data imputation. *CKD: Chronic kidney disease; RD: Rheumatic disease; IJD: Inflammatory joint disease; CTD: Connective tissue disease.
