# Supplementary material for: Socioeconomic factors and COVID-19 mortality in immune-mediated rheumatic diseases patients: regional analysis from Argentina, Mexico and Brazil
Source: Adv Rheumatol. 2024 Oct 9;64:78. doi: 10.1186/s42358-024-00418-3 (PMC13488799; doi:10.1186/s42358-024-00418-3)
Supplement: Supplementary file 3 [file 42358_2024_418_MOESM3_ESM.docx]

Supplementary table 1: Mortality according to COVID-19 diagnostic test

|  | Alive | Dead | p |
| --- | --- | --- | --- |
| n | 4489 | 255 |  |
| Country ,N (%) |  |  | <0.001 |
| Argentina | 2433 (54.2) | 101 ( 39.6) |  |
| Brazil | 1000 (22.3) | 44 ( 17.3) |  |
| Mexico | 1056 (23.5) | 110 ( 43.1) |  |
| Dead = Yes (%) | 0 ( 0.0) | 255 (100.0) | <0.001 |
| COVID-19 presumptive diagnosis (symptoms/epidemiology), N (%) | 587 (13.1) | 19 ( 7.5) | 0.009 |
| COVID-19 RT-PCR, N (%) | 3174 (70.7) | 199 ( 78.0) | 0.012 |
| COVID-19 Antigen test , N (%) | 214 ( 4.8) | 7 ( 2.7) | 0.136 |
| COVID-19 serology, N (%) | 673 (15) | 16 ( 6.3) | <0.001 |
| COVID-19 metagenomic testing , N(%) | 2 ( 0.0) | 0 ( 0.0) | 0.736 |
| COVID-19 lung computerized tomography scan, N (%) | 362 ( 8.1) | 113 ( 44.3) | <0.001 |
| COVID-19 other tests, N (%) | 76 ( 1.7) | 11 ( 4.3) | 0.033 |
