# Supplementary material for: Socioeconomic factors and COVID-19 mortality in immune-mediated rheumatic diseases patients: regional analysis from Argentina, Mexico and Brazil
Source: Adv Rheumatol. 2024 Oct 9;64:78. doi: 10.1186/s42358-024-00418-3 (PMC13488799; doi:10.1186/s42358-024-00418-3)
Supplement: Supplementary file 4 [file 42358_2024_418_MOESM1_ESM.docx]

**Supplementary figure 1. Patients selection**
